# OFFLU Network on Avian Influenza

**DOI:** 10.3201/eid1208.060380

**Published:** 2006-08

**Authors:** Steven Edwards

**Affiliations:** * Veterinary Laboratories Agency, Addlestone, United Kingdom;; † World Organisation for Animal Health, Paris, France

**Keywords:** Influenza in Birds, Information Services, FAO, United Nations, OIE, World Organisation for Animal Health, dispatch

## Abstract

OFFLU is the name of the network of avian influenza expertise inaugurated jointly in 2005 by the Food and Agriculture Organization of the United Nations and the World Organisation for Animal Health. Achievements and constraints to date and plans for the future are described.

Many bird species are susceptible to infection with influenza A viruses. Aquatic birds form a major reservoir; they harbor representatives of all 16 hemagglutinin subtypes and 9 neuraminidase subtypes (*1*). Most of these viruses are of low pathogenicity, whether in wild or domesticated bird species, and most do not cross-infect mammals. Strains of virus that are highly pathogenic for domestic poultry (chickens and turkeys) emerge from time to time and can cause epidemics with high death rates in affected flocks; clinical signs and pathologic lesions have been described (*2*). These highly pathogenic strains are almost invariably of hemagglutinin types 5 or 7 (H5 or H7), although low-pathogenic strains of H5 and H7 also circulate in wild birds and can infect poultry.

Outbreaks of H5 or H7 highly pathogenic avian influenza have occurred in recent years in different countries, including Italy, the Netherlands, Canada, and Mexico, but the most notable global event has been the progressive spread of highly pathogenic H5N1 virus, originally in Asia, then extending into parts of Europe and Africa. A feature of this virus is its ability to infect and kill human hosts, although so far it appears not to have spread substantially from human to human, apart from some possible family clusters. This spread has led to the mobilization of World Health Organization (WHO) and medical virology forces worldwide to monitor the perceived threat of a potential human pandemic (*3*).

For all its importance as a zoonotic threat, highly pathogenic avian influenza is a devastating disease of domestic poultry, which is one of the world's primary sources of animal protein, and the economic effects on livestock producers and rural communities in affected countries are enormous. All strains of H5 and H7 avian influenza viruses, plus any other H subtypes that show pathogenic traits in poultry, are formally notifiable by national veterinary authorities to the World Organisation for Animal Health (OIE) (*4*). Highly pathogenic avian influenza is among the transboundary animal diseases, which are a major concern for international organizations involved in disease surveillance and control, namely OIE and the Food and Agriculture Organization of the United Nations (FAO) (*5*).

Recognizing the global threat posed, particularly by the H5N1 epidemic, the international organizations OIE and FAO agreed in 2005 to establish a network of expertise to support international efforts to monitor and control this disease in poultry and other bird species. The network was designed from the start to interface with the existing WHO influenza network, which was focused on the threat to human health. The new animal influenza network was named OFFLU.

The originally stated objectives of OFFLU were to develop research on avian influenza, offer advice and veterinary expertise to member countries, and collaborate with the WHO animal influenza network. In the year since the network was established, the highest priority tasks have become exchanging scientific data and virus isolates (both within OFFLU and in liaison with the WHO network) and providing experts to assist with missions to affected countries. Developing research activities remains an essential need and will be pursued by individual participant institutions, either alone or in partnerships, but it is not the highest priority task for OFFLU itself.

The core of OFFLU is its scientific committee. Its members represent most of the world's expertise on avian influenza. The activities of the committee and the network are directed by a steering committee that represents key interests of the partner organizations, FAO and OIE. The network is supported by a secretariat, currently located at Padova, Italy, at an OIE/FAO reference center on avian influenza. The network has a philosophy of openness and is keen to involve scientific collaborators from as wide a field as possible. The core network is built around the OIE and FAO reference laboratories for avian influenza, but it is not limited to laboratories. Epidemiologists in particular have a role to play. Qualified persons and institutions are invited to register with the network as scientific collaborators. In addition, the network seeks to establish links with field experts with knowledge and experience of the global poultry industry and the control of infectious diseases, as well as with ornithologists and experts in wildlife diseases.

The relevance of OFFLU was recognized at the Beijing avian influenza pledging conference (*6*). However, funding is needed to empower and sustain this network. Without specific funding, its effectiveness will be limited. Veterinary and scientific expertise on avian influenza is in short supply given the scale of the current epidemic. Requests to FAO and OIE for expert missions to affected countries arrive on a regular basis, and demand exceeds supply. By establishing and extending the network, we hope that these pressures can be mitigated and needs, particularly for developing countries, can be adequately met. OFFLU itself does not send missions, but it provides a cadre of expertise to organizations that undertake this task.

The current priorities of OFFLU are to promote the collection, exchange, and characterization of animal influenza viruses, including the deposition of sequence data in genome banks. A good start has been made on this process, and it is a priority for further development. In addition, the network will seek to establish linkages between laboratories in industrialized and developing countries to provide capacity building and training. The efforts to supply consistent, coordinated advice, expertise, and technical assistance to infected countries will continue. Research will continue on numerous fronts and will address scientific questions such as the molecular basis for virulence, factors involved in host specificity, basic and applied immunology including vaccine development, epidemiology, evaluation of control strategies, and the development of better diagnostic tests.

The network is facing several constraints, including heavy demand on limited resources, regulatory issues (e.g., problems with shipping viruses between countries), and the inevitable tensions between sharing information as a public good versus the desire to protect intellectual property rights. But after only 1 year in operation, the OFFLU network is already proving its effectiveness. It has an active website (http://www.offlu.net) as a primary means of communication, and it is well placed to channel the world's limited scientific resources to best effect in tackling this disease, which is of such great international concern for both animal and human health.
